# Jaw Manipulation Using Ultrasound-Guided Inferior Alveolar Nerve Block for Trismus in Temporomandibular Disorders

**DOI:** 10.1155/2022/4436893

**Published:** 2022-02-26

**Authors:** Yuki Kojima, Ryozo Sendo

**Affiliations:** ^1^Department of Anesthesiology, Asahi General Hospital, Chiba, Japan; ^2^Department of Anesthesiology, Imakiire General Hospital, Kagoshima, Japan

## Abstract

Temporomandibular disorders are a group of disorders with symptoms that include pain and clicking sounds in the temporomandibular joint and restricted mouth opening. For the treatment of temporomandibular disorders with trismus, herein, we suggest a new approach: “jaw manipulation using the ultrasound-guided inferior alveolar nerve block technique.” A woman in her 60s developed temporomandibular disorders and presented with severe trismus owing to pain in the temporomandibular joint. Ultrasound-guided inferior alveolar nerve block was performed with ropivacaine, which relieved the pain in the patient. Furthermore, we performed jaw manipulation for trismus. Since the analgesic effect lasts for 3 days, self-training can be performed while the pain is relieved. After five sessions of “jaw manipulation using the ultrasound-guided inferior alveolar nerve block technique,” trismus significantly improved in this patient. Ultrasound-guided inferior alveolar nerve block can be effective in relieving temporomandibular disorder-related pain and trismus.

## 1. Introduction

Temporomandibular disorder (TMD) is a collective term for functional and structural disorders involving the temporomandibular joint (TMJ), masticatory muscles, head and neck muscles, and/or related tissue components [1, 2]. Its symptoms include pain and clicking sounds in the TMJ and restricted mouth opening, with pain being the most common symptom and frequent reason for seeking treatment [3–5]. Several anatomical, biological, behavioural, biomechanical, and emotional factors affect the masticatory system, contributing to the development of signs and symptoms and/or perpetuation of TMDs. Therefore, TMDs can be considered multifactorial diseases [4]. Restricted jaw movement causes trismus, which leads to difficulty in eating and receiving dental treatments. If the symptoms do not improve with treatment, the patient's mental health can be adversely affected. Moreover, if the condition becomes chronic, the symptoms may become too severe to manage and improve. Therefore, the effective treatment of TMDs associated with trismus can significantly improve the quality of life [1, 5].

The manipulation of the jaw has been the cornerstone of TMD treatment. However, this procedure elicits severe pain in patients with chronic TMD. Ultrasound-guided trigeminal nerve block has been administered for perioperative analgesia in maxillofacial surgery [6, 7]. Ultrasound-guided inferior alveolar nerve block (IANB) with ropivacaine achieves effective long-term pain relief in the trigeminal nerve's region of distribution. We hypothesized that the use of ultrasound-guided IANB would facilitate painless jaw manipulation.

Herein, we report the case of a patient who underwent a novel approach for treating severe trismus due to TMDs.

## 2. Case Presentation

A woman in her 60s presented with a medical history of hypothyroidism, rheumatoid arthritis, and ulcerative colitis. Six months prior to presentation, she had experienced severe pain when she attempted to open her mouth wide. Subsequently, she had been experiencing pain on mouth opening wide. Physical as well as imaging examinations did not reveal any abnormality in the morphology of the TMJ. Bite splint therapy did not result in any improvement, and the intensity of pain prevented training for mouth opening. She was referred to an outpatient pain clinic, where five trigger point local anaesthesia injections were administered, but without any improvement. Furthermore, Chinese herbal medicine did not alleviate her symptoms. Therefore, she was referred to our clinic, which specialises in dental anaesthesiology.

When pain on mouth opening is relieved, the fear of opening the mouth to eat is reduced. To evaluate the effect of treatment, the maximum mouth opening and visual analogue scale (VAS) scores were measured for the quantification of pain. Since TMDs are also associated with emotional factors, we used the hospital anxiety and depression scale (HADS) scores for her mental evaluation (Table 1).

**Figure 1:**
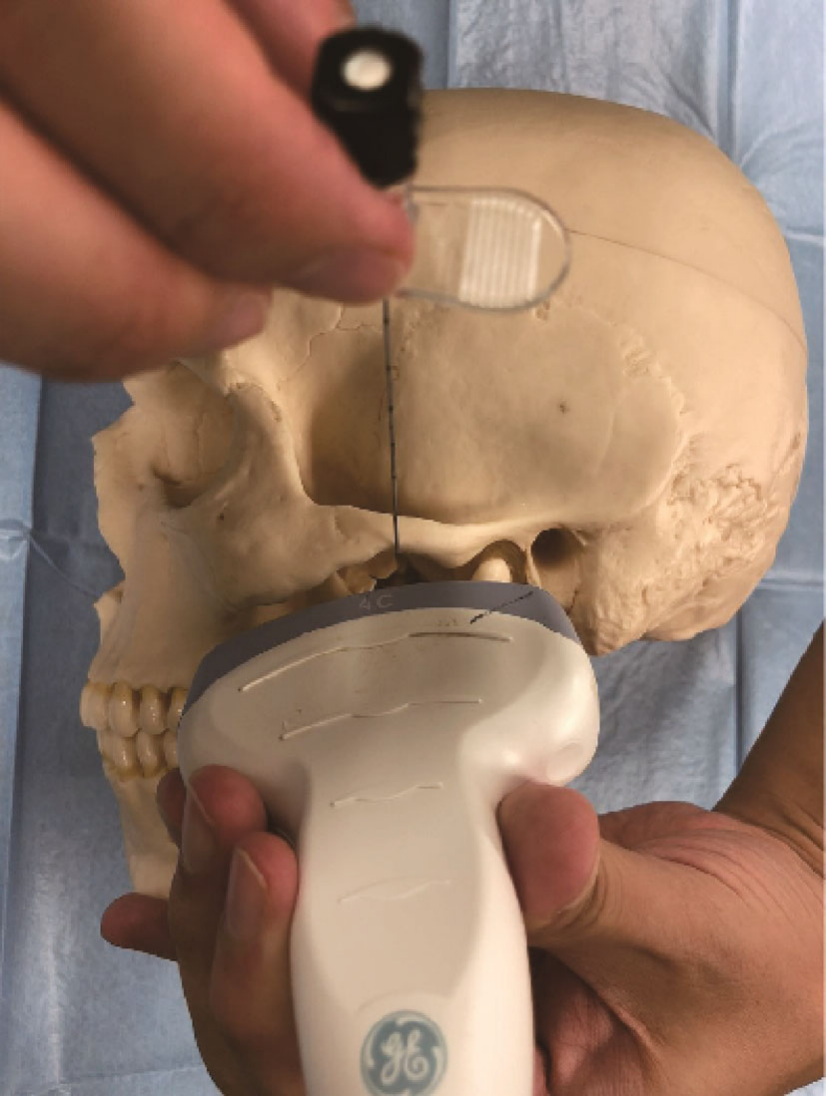
The technique of ultrasound-guided inferior alveolar nerve block.

**Figure 2:**
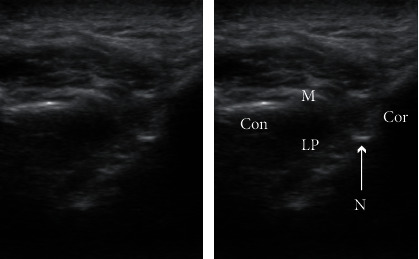
Intraprocedural image of ultrasound-guided IANB. We confirmed the spread of the local anaesthetics (6 ml of 0.375% ropivacaine). Cor: coronoid; Con: condyle; M: masseter; LP: lateral pterygoid muscle; N: needle.

**Figure 3:**
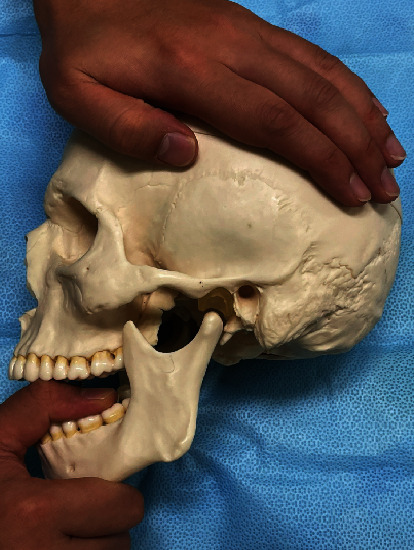
The technique of jaw manipulation.

The patient was treated using this novel approach. The patient was seated in the dental chair, and the vital signs were verified to be normal. The head was turned away from the clinician, and a linear ultrasonic probe was placed just caudad to the zygomatic arch. Thereafter, the masseter and lateral pterygoid muscles were observed. The space between them is the pterygomandibular space (PMS), which contains the inferior alveolar nerve and the peripheral branches of the mandibular nerve [8]. A 22 G needle was inserted to reach the PMS near the inferior alveolar nerve using the extraoral approach (Figure 1). Ultrasound-guided IANB was performed bilaterally with 6 ml of 0.375% ropivacaine on each side. An ultrasound can confirm the spread of the local anaesthetic (Figure 2), and the needle can be inserted while avoiding blood vessels [6]. Jaw manipulation was performed using the conventional method [9]. The patient's forehead was held to stabilise the head, and the thumb was placed on the mandibular posterior teeth. Thereafter, the patient was instructed to open the mouth with maximum effort while being aided in this movement (Figure 3). We have designated this method the “jaw manipulation using the ultrasound-guided IANB (JMUI)” technique. Since the analgesic effect lasts for 3 days, self-training can be performed in the absence of pain. The patient was instructed to use her fingers for self-training in opening the mouth.

**Figure 4:**
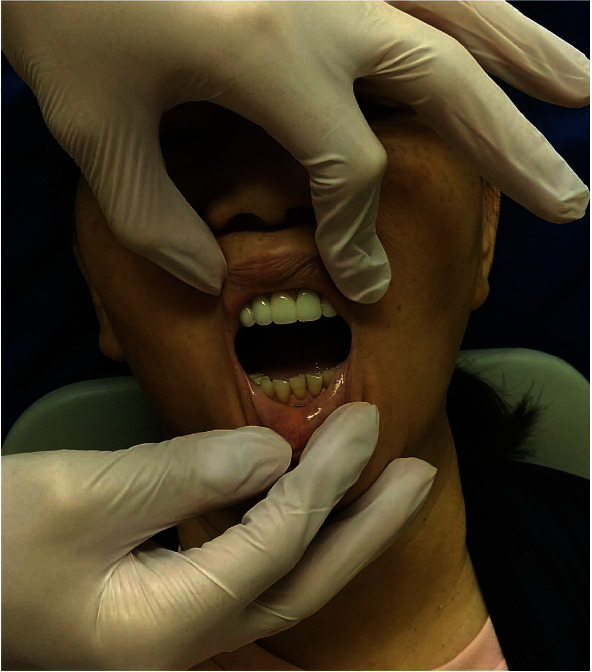
Before the JMUI technique.

**Figure 5:**
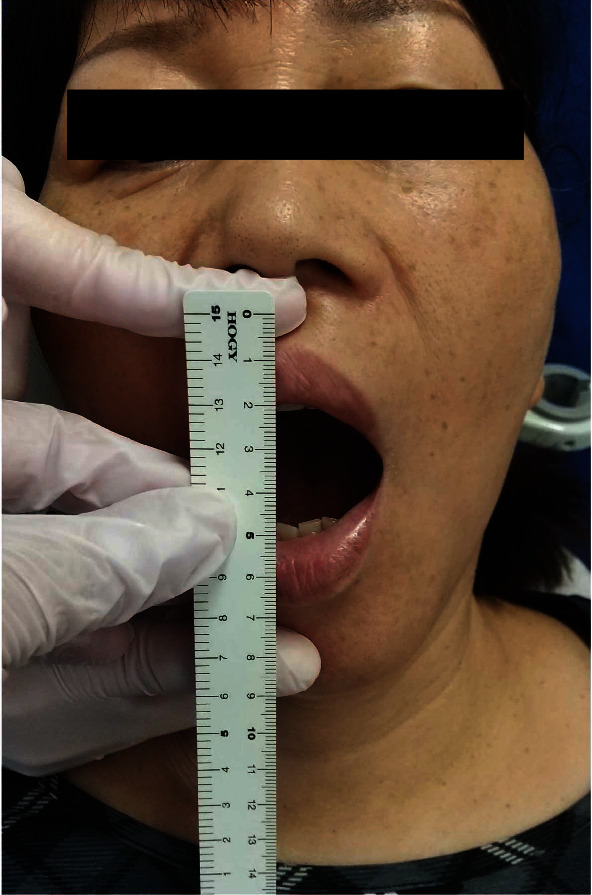
After the first session of JMUI technique.

At presentation, her maximum mouth opening was 15 mm, and the pain on wide mouth opening was assigned a VAS score of 100/100 (Figure 4). Since no morphological changes were evident in the TMJ, pain in the masticatory muscles and disc dislocation was suspected. Hence, we applied the “JMUI technique.” The maximum mouth opening increased to 28 mm, and the pain reduced to a VAS score of 21/100 (Figure 5). The procedure was performed once a week, and after 5 weeks, the maximum mouth opening was 40 mm and the VAS score was 21/100. The HADS score was also improved significantly (Table 1).

## 3. Discussion

The normal range of mouth opening varies individually, but a maximum mouth opening of 38-40 mm is required for the normal functioning of routine activities. Our patient had a chronic TMD. All standard treatments for TMD were administered; however, there was no improvement in pain when opening the mouth. The trismus persisted due to severe pain on mouth opening.

Ultrasound-guided IANB alleviated the trismus caused by TMD-related pain in our patient, who exhibited satisfactory recovery after five sessions. Furthermore, the training for mouth opening was continued (daily, 30 min/day), and maximum mouth opening was maintained. No complications were reported. A recent study showed that ultrasound-guided IANB is useful in the perioperative management of patients who undergo mandibular sequestrectomy for medication-related osteonecrosis of the jaw [6]. The results demonstrated that IANB provides effective pain control for 72 h. In our case, the improvement in mouth opening lasted for 3 days after the procedure, which is consistent with the previous report.

The “JMUI technique” may be effective in cases wherein the traditional treatment modalities are unsuccessful. Jaw manipulation and home training for mouth opening are difficult in patients who experience severe pain during mouth opening. The pain hinders self-motivation for treatment. The “JMUI technique” has the advantage of achieving a longer duration of analgesia than other methods. If the pain is relieved, self-motivation for treatment can also be improved. It is noteworthy that mental stress is also an exacerbating factor for TMDs. Therefore, we evaluated the associated mental factors using the HADS score. In our patient, these scores improved after five sessions. Negative cognition can be corrected by making the patient aware that the pain has been relieved and mouth opening has been increased by the JMUI technique, thus highlighting the technique's therapeutic effect. Such novel treatment methods improve mouth opening and reduce pain, ultimately addressing anxiety and depression-related pain. TMDs can be treated by addressing mental stress, as it is an important contributing factor. Additionally, ultrasound can also be used for blocking the masseteric nerve, which can relax the muscles of mastication. Aside from pain relief, addressing their mental health is equally important.

This approach to clinical practice can apply to refractory severe chronic TMD, even if dentists have provided an effective treatment. As it is less invasive and reversible than surgery, it may be administrable earlier than conventional treatments. This technique was performed by a pain clinician, dentist, or anaesthesiologist, who requires specific training for administering nerve blocks with ultrasound devices.

Because this is a case report, we cannot conclude that the technique would be effective in all types of TMDs. Further long-term studies with a greater number of participants are needed to clarify its definitive indications and effectiveness.

## 4. Conclusion

The JMUI technique involves jaw manipulation after ultrasound-guided IANB. This technique may be a novel approach for the effective treatment of trismus due to chronic TMD. After undergoing JMUI, self-training can be performed while the pain is relieved. Moreover, after five sessions, trismus remarkably improved in our patient.

## Figures and Tables

**Table 1:** Changes in the maximum mouth opening, visual analogue scale, and hospital anxiety and depression scale scores.

	The maximum mouth opening (mm)	Visual analogue scale	Hospital anxiety and depression scale
	Case 1	Case 1	Case 1
Before treatment	15	100/100	24
After 1st treatment	28	21/100	—
After 2nd treatment	30	—	—
After 3rd treatment	30	—	—
After 4th treatment	33	—	—
After 5th treatment	40	21/100	9
